# Screening Adult Patients in Emergency Departments (EDs) for Risk Factors of Child Maltreatment. A Swedish Pilot Study

**DOI:** 10.1111/apa.70703

**Published:** 2026-07-22

**Authors:** Christoffer Dovik, Josefine Cattell, Lisbeth Jönsson, Andreas Rantala, Pia Lundqvist, Louise Laurell

**Affiliations:** ^1^ Department of Internal Medicine and Emergency Care Skåne University Hospital Malmö Sweden; ^2^ Department of Paediatrics Skåne University Hospital, Lund University Lund Sweden; ^3^ Integrative Health Research, Department of Health Sciences Lund University Lund Sweden; ^4^ Department of Paediatrics, Clinical Sciences Skåne University Hospital, Lund University Lund Sweden

**Keywords:** child maltreatment, emergency department, mandatory report of concern, risk factors, screening instrument

## Abstract

**Aim:**

Swedish healthcare staff are obliged to immediately report suspected child maltreatment (CM) to child protection services (CPS), but often fail this duty. The use of screening instruments in EDs increases detection of adults with risk factors for CM. Such instruments have previously not been available in Sweden. The aim of the present study was to pilot test a Swedish version of the Dutch ‘child‐check’ instrument, and evaluate a retrospective case series.

**Methods:**

Our study was performed in an ED at Skåne University Hospital. The original Dutch instrument was translated with adaptation to national medico‐legal context; face validity was obtained by a multidisciplinary expert panel, and it was piloted by a multiprofessional ED team. Following implementation, retrospective data was collected 2021–2022 from registries at the hospital ED and one municipal CPS.

**Results:**

The screening instrument identified 295 adult care‐seekers with risk factors for CM. Subsequent reports of concern included 131 children, among which 62 (47%) were not known to CPS.

**Conclusion:**

Our study suggests that the Swedish version of the instrument, ‘child‐check adult’, could play an important role for ED personnel in identifying adults with risk factors for CM. We therefore aim to implement it in all Swedish regions.

## Key Notes

- A Dutch instrument for targeted clinical‐based screening of CM was piloted in a Swedish ED. It identified risk factors in 295 adult care‐seekers, resulting in reports of concern for 131 children.
- Sixty‐two (47%) of the children were previously not known to CPS, suggesting that the new Swedish instrument could play an important role in detecting children at increased risk of harm.
- We suggest further research to explore ED and CPS staff's perceptions.

**Abbreviations**
CM: Child maltreatment
CNI: Care need index
CPS: Child protection services (Socialtjänst in Swedish)
ED: Emergency department
EDIS: Emergency department information system
EHR: Electronic health record
RETTS: Rapid emergency triage and treatment system
UN: United Nations
WHO: World Health Organisation

## 1 Introduction

Child maltreatment (CM) was defined by WHO as ‘the perpetration of physical, sexual and psychological/emotional violence or neglect of infants, children and adolescents aged 0–17 years by parents, caregivers and other authority figures’ [1]. A review of prevalence studies from 96 countries showed that more than half of all children aged 2–17 years had experienced emotional, physical or sexual violence in the past year [2]. Reports from high‐income countries estimate that 4%–16% of children are physically abused and that one in 10 are exposed to neglect or psychological abuse every year [3]. Thus, CM is a prevalent and major public health and social welfare problem [3, 4].

The UN Convention on the Rights of the Child was ratified by Sweden in 1990 and became statutory law in 2020. It states that ‘every child has a right to the highest attainable standard of health and a childhood freed from violence and neglect’ [5]. Swedish law mandates immediate report for healthcare professionals to child protection services (CPS) on every suspicion of child abuse or neglect. In contrast to more litigious child protection systems, the family‐service‐oriented Swedish model prioritises preventive interventions, and a defining feature is a low threshold for reporting concerns. CPS reports are submitted to the local municipality of residence. Unfortunately, surveys show that staff often fail to fulfil this obligation [6].

Emergency departments (EDs) must cope with large patient volumes, and the staff often face working conditions with short patient encounters and limited time for reflection [7]. Basic information regarding presence of children in the household or presence of domestic violence is seldom systematically requested [8, 9]. While machine learning models for predicting CM are in development, they cannot yet replace human‐led screening based on clinical observation of risk factors [10].

With the aim of piloting a Dutch model identifying adults with risk factors associated with CM, a collaboration was established in 2017 with Dutch researchers and originators of the ‘child‐check’ instrument, originally named The Hague protocol. It serves as a clinical decision support tool for ED staff to systematically identify parents with risk factors that indicate an increased risk of CM. The Netherlands, with a comparable socioeconomic profile to Sweden and a similar healthcare system, mandates the use of CM screening instruments for ED staff. The facilitators and barriers for the approach have been scientifically mapped, and the ‘child‐check’ instrument is now implemented nationwide [7]. The first step of the screening procedure is to ask if an adult, presenting with their own medical problems, is a *parent*. If yes, the second step is to screen for any of three risk factors: *domestic violence, substance abuse and severe mental health disorder* [11, 12]. Following its introduction in the Netherlands, the number of referrals to the Reporting Center for Child Abuse and Neglect (RCCAN) increased from 1 to 64 per 100 000 adult ED patients. After assessment by RCCAN, CM was confirmed in 91% of the cases [12].

## 2 Material and Methods

The Swedish version of the screening instrument was based on the original Dutch ‘child‐check’ instrument. With permission from the originators it was translated and adapted for the Swedish context in 2019. Translation, cultural adaptation and face validation were performed according to the WHO cultural adaptation guidelines [13]. Forward‐translation of the original Dutch document to Swedish was conducted by the senior author (L.L.) with extensive Swedish experience in child abuse paediatrics, in collaboration with a Dutch originator of the instrument. Subsequently, a Dutch native speaking Swedish doctor performed a backward translation. The cross‐linguistic comparison underscored cultural and linguistic differences in how the concept of illicit drug use was perceived. The Swedish version was then reviewed by three expert panels. These included a multidisciplinary child protection team (paediatrician, specialist paediatric nurse, social worker), a multiprofessional group of ED professionals (registrar in emergency care medicine, specialist nurse in emergency care, medical secretary and social worker), and social workers from the municipal CPS.

### 2.1 Setting

The final Swedish version was called ‘child‐check adult’ and fully implemented in 2021 at the ED of Skåne University Hospital in Lund, Sweden. The ED serves a primary catchment area with approximately 300 000 inhabitants. The patient population includes both walk‐ins via triage and ambulance arrivals. As a designated regional level 1 trauma centre, a tertiary academic medical centre and a referral centre in southern Sweden, the hospital provides specialised care for approximately 1.9 million inhabitants. A social worker on duty is available on‐site during weekday business hours, and on‐call during off‐hours.

The multiprofessional ED working group continuously assessed that screening procedures and patient workflow remained functional. Implementation and evaluation of ‘child‐check adult’ were conducted in accordance with evidence‐based national guidelines and checklists from The National Board of Health and Welfare and The Public Health Agency of Sweden [14, 15]. To facilitate the process, members of the research team, the child protection team, the multiprofessional group of ED staff, and social workers from CPS met for stakeholder dialogues. Meetings were held prior to and during implementation, with detailed written records for each session.

### 2.2 Procedure

The procedure was finalised in several steps:

- Initially all ED staff were reminded of their statutory duty to immediately report suspected CM to CPS.
- The screening protocol was integrated into the mandatory introduction training for all new nurses, assistant nurses, and physicians.
- The initial screening question, whether the patient is a caregiver for or lives in a household with children under 18, was added as a mandatory field to the RETTS (Rapid emergency triage and treatment system) paper chart.
- If a child was present in the household and any of the three predefined risk factors was met: (1) domestic violence, (2) substance abuse and (3) severe mental health disorder, a formal report to CPS was initiated. Screening was carried out 24 h a day, 7 days a week. While the triage nurse identified the presence of children, the assigned nurse and physician were responsible for carrying out the screening protocol. When submitting a report, ED staff were advised to routinely provide a leaflet to their patient: ‘Information’ to caregivers for children: Report of concern to CPS, available in 9 different languages.

Because the current Electronic health record (EHR) could not include mandatory checklists, screening was conducted using paper‐based forms. Due to the absence of a digital reporting interface, medical secretaries sent paper forms of reports of concern to the CPS department concerned by post or fax.

### 2.3 Data Collection and Analysis

Retrospective data collection covered a two‐year period from January 2021 to December 2022.

Anonymised data on patients reported to CPS due to positive screening was extracted from the electronic ED information system (EDIS). Retrieved data was as follows:

- Age of adult care‐seeker.
- Gender (male or female, according to the national population register).
- Identified risk criteria.

Visitor numbers and residential municipalities for visitors to the Lund ED were recorded for 2021 and 2022. Socio‐demographic information, measured as the Care need index (CNI, Appendix S1), was obtained for all municipalities accounting for at least 1000 patient visits in 2021.

Registry data concerning specific outcomes of ‘child‐check’ screening was collected from Lund CPS, the municipality where a significant portion of healthcare‐seeking individuals attending the Lund ED reside. Anonymised data available was:

- The number of child‐check adult reports received
- The number of children identified
- Whether CPS had prior knowledge of the child or not

Quantitative data were analysed at a descriptive level.

## 3 Results

### 3.1 Adaptation, Face Validation and Implementation of the Screening Instrument

Implementation and evaluation of the screening instrument adhered to evidence‐based guidelines and checklists [14, 15]. Key medico‐legal, cultural, and linguistic differences between Sweden and the Netherlands were discussed within the expert panels, and suggested modifications were inserted to better align with a Swedish context.

The expert panels agreed that, based on Swedish legislation, the presence of one or more risk factors should always trigger an immediate report to CPS. The expert panels also concluded that there was a need to broaden the initial Dutch screening question ‘if the adult is a parent’, to ‘all legal guardians and adults in regular contact with children in the household regardless of guardianship status’.

When it came to the three predefined ‘child‐check’ criteria it was agreed that the definition of *substance abuse* needed to be aligned with Swedish legislation. Misuse of alcohol, narcotics, illegal drugs and prescription drugs was considered equivalent.

Domestic violence was defined as any form of violence occurring within the household where the victim has a close relationship with and emotional ties to the perpetrator. Inquiries regarding violence were initiated upon clinical suspicion of secondary symptoms or signs.

*Severe mental illness* was established as the threshold for mental health concerns, and was further defined as either encompassing psychosis, serious self‐harm, and/or suicide attempt.

Field testing of ‘child‐check adult’ was performed in the ED setting for 3 months, in continuous dialogue with CPS. Based on feedback from CPS and the multiprofessional ED team, minor adjustments to the screening procedure and the inter‐agency cooperation were made prior to the start of implementation:

- Due to privacy‐related restrictions on accessing information about children in the household, new guidelines were issued in conjunction with the hospital's legal department to govern collection of such non‐disclosable information in unconscious/unresponsive patients
- A revised and abbreviated report of concern notification form was developed, focusing on the essential information required for the CPS decision‐making process.
- A shared inbox was created to handle email queries from CPS related to ‘child‐check’ screening and subsequent reports of concern.

During implementation, regular multidisciplinary feedback sessions were held to present the number of reports generated and to address facilitators and barriers, such as high workload and challenges imposed by the Covid‐19 pandemic.

### 3.2 Outcome

Data was only available for patients for whom a positive screening result was recorded. During the two‐year study period, 295 adults with children in their households and with at least one of the three risk criteria were identified in the EDIS.

Figure 1 illustrates the primary risk criterion of reports and the gender distribution. It was slightly more common that women were identified and reported to CPS, 56% for women and 44% for men. The dominating risk criterion in both women and men was substance abuse, accounting for 72% of reports. Domestic violence and severe mental illness accounted for 14% each.

**FIGURE 1:**
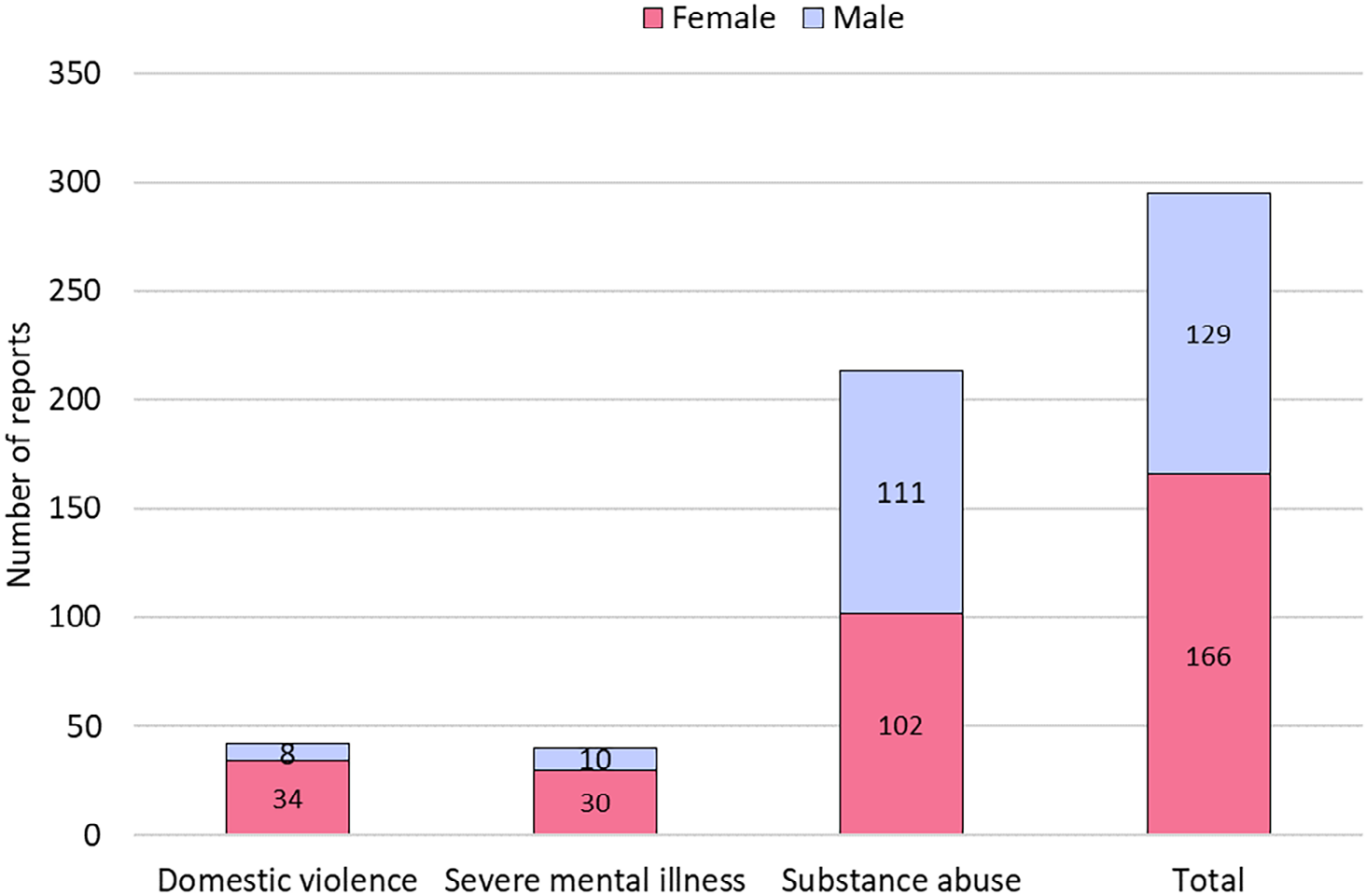
Reports to child protection services according to ‘child‐check adult’ criteria and gender (*n* = 295). The primary risk criterion of 295 reports during 2021 and 2022, and the distribution of reports by gender of the care‐seeking adult.

Figure 2 shows the primary risk criterion in reports across different age cohorts. Sixty‐four % of reports concerned patients between 31 and 50 years of age, but ages of reported adults ranged from 18 to 82 years (18–81 for women and 18–82 for men), with a mean of 38 years.

**FIGURE 2:**
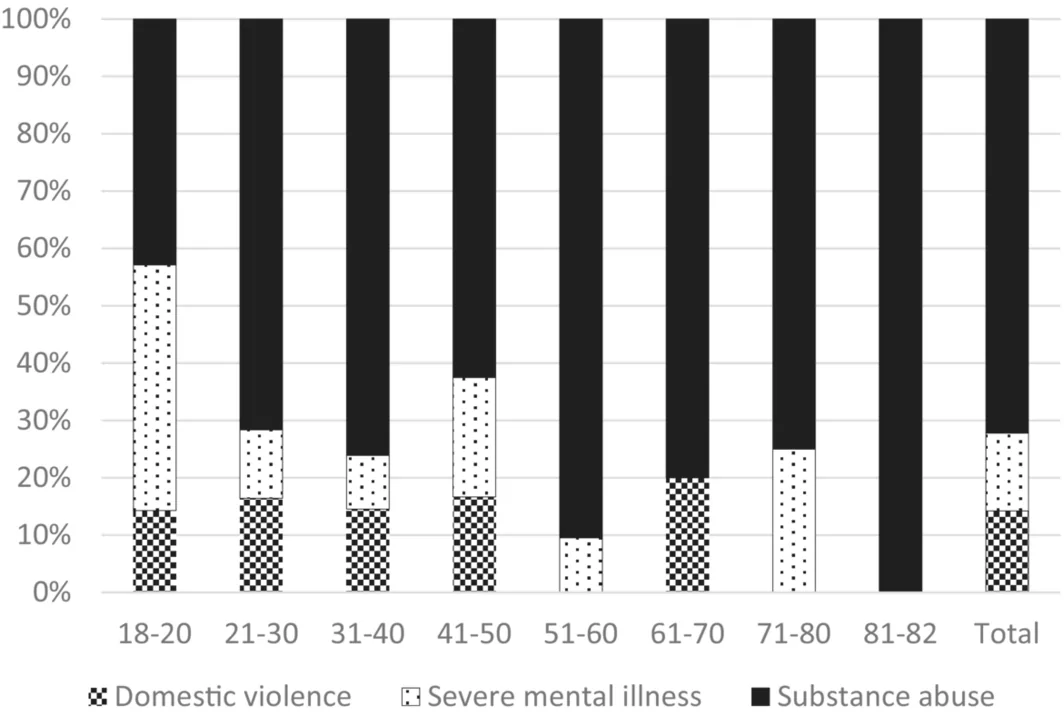
Reports to child protection services according to ‘child‐check adult’ criteria and age (*n* = 295). The primary risk criterion of 295 reports during 2021 and 2022, across different age cohorts of care‐seeking adults.

A total number of 114 359 patients visited the ED during 2021–2022. Data regarding the total number of individuals screened was not available. In cases where multiple risk criteria were registered in the screening protocol, the presenting condition for the ED visit was used to select a primary criterion. Table 1 presents sociodemographic conditions, defined by the CNI, in the 11 municipalities where 94% of the patients seeking care in 2021 were registered residents.

**TABLE 1:** Municipality of residence and sociodemographics.

Municipalities (*n* = 11)	ED patients, *n* (%)	Inhabitants (*n*)	Care need index^a^
Lund	19 595 (34)	127 376	1.019
Eslöv	5893 (10)	34 593	1.015
Kävlinge	5265 (9)	32 341	0.775
Landskrona	4219 (7)	46 488	1.134
Staffanstorp	3968 (7)	26 242	0.789
Lomma	3869 (7)	24 638	0.628
Hörby	2718 (5)	15 745	0.915
Burlöv	2676 (5)	19 753	1.290
Höör	2601 (4)	16 954	0.820
Svalöv	1591 (3)	14 412	0.951
Malmö	1482 (3)	351 749	1.459
	Total 53 877 (94)	Total 710 291	Mean 0.981

*Note:* Sociodemographic factors, as measured by the Care need index, in the 11 municipalities accounting for 94% of all healthcare‐seeking individuals attending Lund ED in 2021.

^a^ The Care need index (CNI) is a standardised socio‐economic tool used in the Swedish healthcare system to predict the expected need for healthcare services in a certain population. CNI serves as a proxy for socio‐economic pressure, and a basis to determine allocation of public funding. An area is compared to the Swedish national average, which is set at 1.0. CNI > 1.0: The population has a higher‐than‐average predicted need for care. CNI < 1.0: The population has a lower‐than‐average predicted need for care. The predefined variables of CNI are presented in Appendix S1. https://www.scb.se/en/services/ordering‐data‐and‐statistics/orderering‐regional‐statistical‐products/care‐need‐index‐cni/.

Figure 3 presents anonymised data from the CPS registry on the outcome of 75 ‘child‐check adult’ reports sent to Lund, the municipality contributing with 34% of healthcare‐seeking individuals. Almost half of the 131 children identified were not previously known to CPS. The remaining 220 reports were sent to other Swedish municipalities.

**FIGURE 3:**
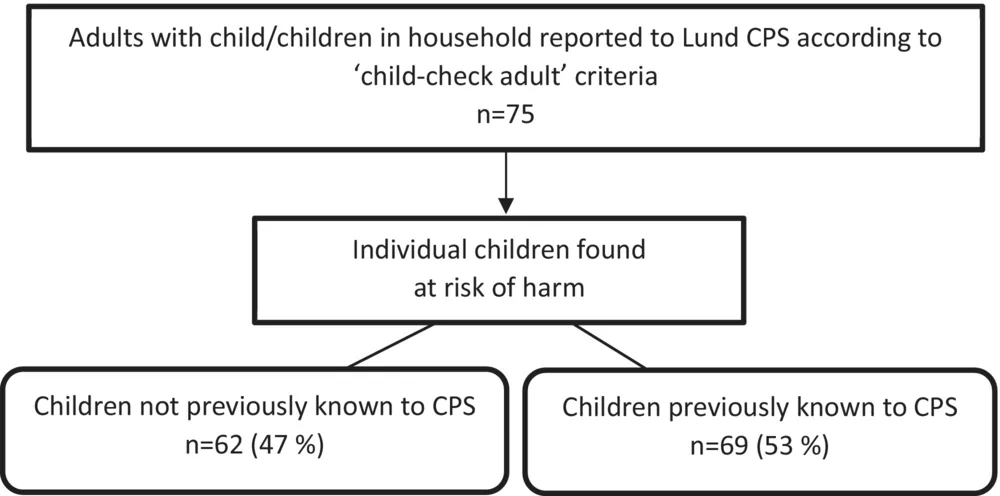
The outcome of ‘child‐check adult’ reports to Lund child protection services (*n* = 75). Seventy‐five reports during 2021 and 2022 concerned 131 individual children, among which 62 (47%) were not previously known to child protection services (CPS).

## 4 Discussion

Our pilot study is part of a broader initiative to implement ‘child‐check’ targeted clinical‐based screening instruments across all age groups in Swedish EDs. It is the first in the Nordic countries to evaluate a tool designed to detect risk factors for CM among adult patients, aligning with the Swedish national strategy to prevent violence against children.

Given the high prevalence of CM and documented gaps in mandatory reporting, identifying children previously unknown to CPS is a significant finding. However, the risk of false positives is a key consideration; a lack of prior contact with CPS does not inherently confirm that a child is at increased risk. While healthcare providers must report suspicions of risk, the formal determination of CM remains the responsibility of CPS. During the implementation process CPS staff noted that the new screening instrument facilitated their work, despite an increased influx of reports. They also highlighted that ‘child‐check adult’ screening identified risks involving adults who were not legal guardians or registered co‐residents at the child's listed address—such as divorced parents with shared custody, step‐parents or grandparents—who nonetheless had routine contact with children (unpublished data). The wide age distribution of adults with identified risk factors in our study underscores the need to systematically assess all adult ED patients, regardless of age or legal guardianship, to ensure that at‐risk adults in extended families are not overlooked. The multidisciplinary ED working group continuously evaluated discrepancies between professional judgement and the initial clinical‐based inquiry. A CPS report was mandatory if any staff voiced concern.

CM may lead to an array of both short‐ and long‐term physical, psychological and behavioural problems, i.e., obesity, chronic pain, depression, suicide attempts, ischemic heart disease, and respiratory problems [3, 16, 17, 18, 19]. The risk of such adverse outcomes increases with the severity and duration of CM. The more types of abuse a child is exposed to, the greater the risk of poor health outcomes [20]. Risk factors for CM are also often interconnected and tend to cluster within families [21]. Despite its high prevalence and negative impact, CM often goes undetected and is grossly underestimated in official reports emerging from law enforcement, social welfare‐ and healthcare organisations [3]. To ensure that children exposed to or at risk of CM receive the support they are entitled to, they must first be detected [22]. General EDs, with their high patient volumes, may play a crucial role in identifying adults who exhibit risk factors associated with an increased risk of CM. Early detection of CM will allow the provision of appropriate assistance, prevent further abuse, and reduce long‐term negative effects [4, 23, 24, 25, 26, 27].

Screening adults for risk factors that can negatively impact children's health and increase the risk of CM is a key preventive measure, though concerns exist that it may discourage adults from seeking essential medical care [28, 29]. However, the authors of the original Dutch ‘child‐check’ instrument found no evidence of such deterrent effect, particularly when explanatory leaflets were used to mitigate patient concerns [29]. In the Swedish context, where public trust in healthcare is high, our staff routinely provided leaflets clarifying the mandatory reporting obligation while emphasising the patient's right to ‘good care and to being treated well, regardless of whether a report has been made’.

Several systemic barriers hampered data collection during this pilot. Registration deficiencies prevented an accurate count of the total numbers of individuals screened. Furthermore, the absence of a standardised digital interface between healthcare and CPS, coupled with non‐uniform documentation practices across Swedish municipalities, made it impossible to track the total volume of reports. Only the municipality of Lund specifically categorised reports originating from the ‘child‐check adult’ screening. Given that Lund accounted for over one‐third of the study population and maintains a CNI comparable to the national average, its data provide a representative insight into the screening's impact (Table 1, Figure 3). Finally, strict confidentiality regulations precluded the tracking of individual outcomes or the results of specific CPS assessments.

Although Sweden and the Netherlands share similarities, their legal framework regarding CM differs significantly. In contrast to Sweden, the Netherlands does not have a mandatory reporting obligation. Our evaluation of ‘child‐check adult’ in Lund revealed that 47% of the 131 identified children were previously unknown to CPS, compared with 73% of children (*n* = 565) in a similar study from The Hague. Direct comparisons are however limited by The Hague's fourfold larger population and distinct sociodemographic profile. Notably, substance abuse was the primary concern in a substantially larger proportion of Swedish reports (72%) than in the Dutch study (14%). These disparities likely stem from the more liberal Dutch stance on substance abuse, contrasted with Sweden's restrictive legislation and mandatory reporting requirements [30].

The instrument was implemented during the Covid‐19 pandemic, which significantly impacted family dynamics and ED working practices. High patient volumes and staffing shortages necessitated the redeployment of medical students and staff from other departments to the ED triage unit—factors that likely influenced both implementation and study outcomes. Despite these challenging circumstances, the project continued due to strong support from the ED staff, as demonstrated in an interview study conducted 6 months post‐implementation (non‐published data).

### 4.1 Ethical Considerations

The debate between universal and targeted screening in the ED highlights an ethical tension between child safeguarding and patient autonomy. By employing a targeted clinical inquiry supported by professional judgement grounded in specific and acute clinical triggers (domestic violence, substance abuse, and severe mental health disorder) the ‘child‐check adult’ instrument fulfils the legal obligation to protect at‐risk children, while actively mitigating the familial distress and systemic burden associated with false‐positive reports.

## 5 Conclusions

The Dutch ‘child‐check adult’ instrument was successfully translated, face validated, and piloted in a Swedish ED. The tool proved effective as it identified a significant number of children previously unknown to CPS. The screening protocol will become mandatory when integrated in a forthcoming update of the regional EHR. This will ensure future compliance to the protocol, as well as automated and objective collection of reporting frequency over time. Ongoing research is exploring ED staff's and CPS staff's perceptions of the instrument.

## Author Contributions

**Christoffer Dovik:** investigation, writing – original draft, validation, visualization, writing – review and editing, software, formal analysis. **Josefine Cattell:** writing – original draft, writing – review and editing, formal analysis. **Louise Laurell:** conceptualization, investigation, funding acquisition, writing – original draft, methodology, validation, visualization, writing – review and editing, formal analysis, project administration, supervision, resources. **Lisbeth Jönsson:** writing – original draft, writing – review and editing. **Pia Lundqvist:** writing – original draft, writing – review and editing, formal analysis, supervision, project administration. **Andreas Rantala:** writing – original draft, writing – review and editing.

## Funding

This work was supported by the Swedish National Council for Crime Prevention, Children's Welfare Foundation Sweden, and the ‘Mjölkdroppen’ Foundation.

## Ethics Statement

The study procedure was reviewed and approved by the National Ethical Review Board (dnr 2022‐03098‐01).

## Conflicts of Interest

The authors declare no conflicts of interest.

## Supporting information


**Appendix S1:** Supporting Information.

## Data Availability

The data that support the findings of this study are available on request from the corresponding author. The data are not publicly available due to privacy or ethical restrictions.
