# Supplementary material for: Screening Adult Patients in Emergency Departments (EDs) for Risk Factors of Child Maltreatment. A Swedish Pilot Study
Source: Acta Paediatr. 2026 Jul 22;115(12):2855–61. doi: 10.1111/apa.70703 (PMC13747675; doi:10.1111/apa.70703)
Supplement: Supplementary file 1 [file APA-115-2855-s001.docx]

**Appendix S1**

**The Swedish care need index**

The Care need index (CNI) is a standardised sociodemographic tool used by Swedish regional health authorities to quantify the expected need for healthcare services within a specific catchment area or population. Unlike models based solely on age or prior diagnoses, the CNI focuses on social determinants of health that are known to correlate with increased morbidity and a higher demand for primary care and emergency services.

**Components of the CNI**

The index is calculated using weighted data from seven sociodemographic variables retrieved from national registries (Statistics Sweden):

1. Elderly living alone: Percentage of individuals aged 65 and older living alone
2. Foreign-born: Percentage of individuals born in non-Western countries
3. Unemployed: Percentage of the population (ages 16–64) without employment
4. Low educational level: Percentage of adults (ages 25–64) with only primary school education
5. Single parents: Percentage of households with children under the age of 17 headed by a single parent
6. High mobility: Percentage of the population that has moved into the area within the last year
7. Children under 5: Percentage of the population aged 0–4 years

**Interpretation and application**

- Standardisation: The CNI is standardised to a national mean of 1.0
- Scoring: A CNI score higher than 1.0 indicates a higher-than-average predicted need for care due to social vulnerability, whereas a score below 1.0 indicates a more socially stable or affluent population with lower predicted healthcare demands
- Resource allocation: In the Swedish healthcare system, CNI is a primary driver for resource weighting, ensuring that clinics serving high-risk populations receive additional funding to compensate for the increased time and complexity required per patient encounter
